# Supplementary material for: A Critical View of the Application of the APEX Software (Aqueous Photochemistry of Environmentally-Occurring Xenobiotics) to Predict Photoreaction Kinetics in Surface Freshwaters
Source: Molecules. 2019 Dec 18;25(1):9. doi: 10.3390/molecules25010009 (PMC7017383; doi:10.3390/molecules25010009)
Supplement: Supplementary file 1 [file molecules-25-00009-s001.zip › 3.2.4_gcc-4.4.0.zip!/3.2.4_gcc-4.4.0/3.2.4_gcc-4.4.0/doc/octave/html/interpreter/Marker-Styles.html]

Marker Styles - Untitled


Next: Callbacks,
Previous: Line Styles,
Up: Advanced Plotting


---

#### 15.2.6 Marker Styles

Marker styles are specified by the following properties:

`marker`: A character indicating a plot marker to be place at each data point, or `"none"`, meaning no markers should be displayed. `markeredgecolor`: The color of the edge around the marker, or `"auto"`, meaning that the edge color is the same as the face color. See Colors. `markerfacecolor`: The color of the marker, or `"none"` to indicate that the marker should not be filled. See Colors. `markersize`: A number specifying the size of the marker. The default is 1. A value of 2 is twice as large as the default, etc.
